# Pdl1 Is a Putative Lipase that Enhances *Photorhabdus* Toxin Complex Secretion

**DOI:** 10.1371/journal.ppat.1002692

**Published:** 2012-05-17

**Authors:** Guowei Yang, Carmen Sara Hernández-Rodríguez, Michael L. Beeton, Paul Wilkinson, Richard H. ffrench-Constant, Nicholas R. Waterfield

**Affiliations:** 1 Institute of Pathogen Biology, Chinese Academy of Medical Sciences & Peking Union Medical College, Beijing, China; 2 Department of Biology and Biochemistry, University of Bath, Bath, United Kingdom; 3 University of Bristol, School of Biological Sciences, Bristol, United Kingdom; 4 Biosciences, University of Exeter, Penryn, United Kingdom; University of California Los Angeles, United States of America

## Abstract

The Toxin Complex (TC) is a large multi-subunit toxin first characterized in the insect pathogens *Photorhabdus* and *Xenorhabdus*, but now seen in a range of pathogens, including those of humans. These complexes comprise three protein subunits, A, B and C which in the *Xenorhabdus* toxin are found in a 4∶1∶1 stoichiometry. Some TCs have been demonstrated to exhibit oral toxicity to insects and have the potential to be developed as a pest control technology. The lack of recognisable signal sequences in the three large component proteins hinders an understanding of their mode of secretion. Nevertheless, we have shown the *Photorhabdus luminescens* (*Pl*) Tcd complex has been shown to associate with the bacteria's surface, although some strains can also release it into the surrounding milieu. The large number of *tc* gene homologues in *Pl* make study of the export process difficult and as such we have developed and validated a heterologous *Escherichia coli* expression model to study the release of these important toxins. In addition to this model, we have used comparative genomics between a strain that releases high levels of Tcd into the supernatant and one that retains the toxin on its surface, to identify a protein responsible for enhancing secretion and release of these toxins. This protein is a putative lipase (Pdl1) which is regulated by a small tightly linked antagonist protein (Orf53). The identification of homologues of these in other bacteria, linked to other virulence factor operons, such as type VI secretion systems, suggests that these genes represent a general and widespread mechanism for enhancing toxin release in Gram negative pathogens.

## Author Summary

Bacterial pathogens of insects deploy a range of toxins to combat the innate immune system and kill the host. There is significant interest in developing these toxins as candidates for crop protection strategies. To date, transgenic crops expressing *Bacillus thuringiensis* Cry toxins have been used to resist predation by pests. In order to minimize the risk of insect resistance development, current research in crop biotechnology comprises the design of new transgenic plants expressing toxins with different modes of action. The Toxin Complex (TC) gene family first identified in the insect pathogen *Photorhabdus* has received interest as an alternative. It remains obscure how *Photorhabdus* regulates, assembles, and secretes such a large toxin complex. We have identified a small lipase protein, Pdl1, which enhances secretion and leads to the release of the Toxin complex off the bacterial surface. This is of wider significance because TC toxin homologues are also found in a range of human pathogens, such as *Yersinia* in which they have been implicated in human virulence. Furthermore homologues of *pdl* are also seen tightly linked to other virulence loci such as the type VI systems of *Vibrio*. We speculate that this Pdl mediated secretion enhancement system is a widespread and important mechanism used by Gram negative bacterial pathogens.

## Introduction

*Photorhabdus* are Gram-negative members of the Enterobacteriaceae that live in a symbiotic association with entomopathogenic nematodes which invade and kill insects. Upon insect invasion the nematode regurgitates the bacteria which grow within the insect open blood system releasing a plethora of toxins and insecticides, to kill the insect and protect the cadaver from invading microbes, scavengers and saprophytes [1]. One major class of secreted insecticidal toxins are the Toxin Complex (TC) proteins [2]–[3]. They constitute large multimeric protein complexes that have been shown to exhibit oral toxicity to a range of insects including crop pests [4]–[5]. These complexes consist of three protein subunit types; homologues of TcdA TcdB and TccC proteins, from here on referred to simply as A, B and C-subunits respectively [6]. These protein subunits themselves are large with the TcdA1, TcdB1 and TccC5 being 2517 aa (283 kDa), 1477 aa (165 kDa) and 939 aa (105 kDa) respectively. Recent work on a *Xenorhabdus nematophilus* TC suggests that the A (XptA2), B (XptB1) and C (XptC1) subunits are in a 4∶1∶1 stoichiometry respectively [7]–[8]. There is now reasonable evidence to indicate that the A subunit forms a cage-like tetramer of around 1120 kDa which associates with a tightly bound 1∶1 sub-complex of B and C. The A subunits can bind the host cell membranes of insect brush border cells and form a pore facilitating the entry of the BC sub-complex [8]–[9]. The toxic activity of the complex resides in the C-terminal region of the C subunit. The TCs represent a potential target to augment the successful *B. thuringiensis* crystal toxin crop protection technology and have been the subject of significant investment by the agrochemical industry. The availability of a large number of bacterial pathogen genome sequences has revealed that TC toxins are not restricted to *Photorhabdus* and *Xenorhabdus* sp., but are in fact widely distributed [2]. This includes Gram-negative human pathogens such as *Yersinia* and *Burkholderia* and a Gram-positive insect pathogen such as *B. thuringiensis* strain IBL200 (accession NZ_ACNK01000119). Although the role of these TC homologues in most pathogens remains obscure, recent work in *Yersinia pseudotuberculosis* has suggested that homologues of these toxins have been adapted to act upon the mammalian gut [10]. They have also been implicated in mammalian gut colonisation in at least one strain of *Y. enterocolitica* [11].

Some progress has been made in TC research including the reconstitution of TC function through heterologous expression of the three essential subunits, A, B and C, in *E. coli* [3], [8], [12]–[13], expression of partial activity in transgenic plants [14] and the analysis of TC homologues from other species of bacteria [10], [15]. In addition TC toxins have also been heterologously expressed in *Enterobacteria* species which associate with termites as a control strategy [16]. Most recently Lang et al demonstrated the mode of action of certain C-subunit C-terminal domains in the ADP-ribosylation of actin and RhoA [9]. Nevertheless, efforts in understanding the biological context of TC have been hampered by an incomplete understanding of how Gram-negative bacteria are able to assemble and secrete such large multimeric protein complexes.

The *tc* gene homologues are encoded at several different loci in the *Photorhabdus* genome and in strain *Pl* W14, two of these loci, *tca* and *tcd* were shown to be responsible for oral toxicity to *Manduca sexta* larvae [4], [17]. The *tcd* locus is a large pathogenicity island (pai) containing multiple homologues of the A, B and C-subunit genes in tandem [18]. Previously we used RT-PCR and western blotting to demonstrate that *Pl* W14 expresses both the *tca* and *tcd* loci genes during insect infection [19]–[20]. Previous work has shown that the A and B+C-subunits are capable of exhibiting oral toxicity independently when expressed at high levels, yet nevertheless assemble into a far more potent large multimeric complex [8], [21].

**Figure 1:**
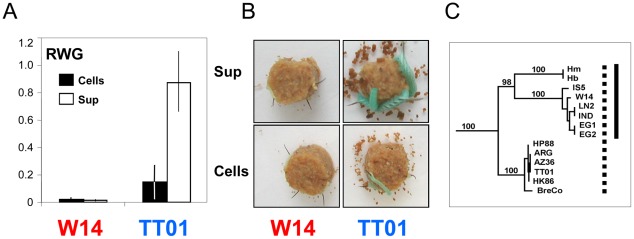
Oral toxicity in *P. luminescens* strains W14 and TT01. (**A**) Oral toxicity of cells (cells) and supernatants (sup) of *P. luminescens* strains W14 and TT01 against *M. sexta* neonates. A cohort of 20 larvae each were tested by oral bioassay on food blocks overlaid with 100 µl of undiluted 3 day old culture supernatants and the equivalent of saline washed cells. Cell numbers added to each block were adjusted to 2×10^8^ cells per block. Relative Weight Gain (RWG), means and standard errors are shown. Data is normalised against an *E. coli* EC100 negative control (which would give a mean weight of 1). (**B**) Illustration of the differential toxicity of cells and supernatants of W14 and TT01 against *M. sexta* neonates after seven days consuming the treated artificial diet. (**C**) *P. luminescens* phylogenetic relationship based on MLST analysis. Note that while all these strains produce the oral toxin Tcd on the cell surface (dotted bar), that those in the clade with strain W14 (solid bar) are also able to release it into the supernatant.

We noted that strains belonging to the species *P. luminescens* could be classified into two distinct biotypes, some produced Tcd which remained attached to the cell surface (e.g. strain *Pl* TT01), while others also released it into the surrounding medium (e.g.; *Pl* W14) (Fig. 1AB). We hypothesized that there would be specific genetic factors facilitating this variation in TC deployment. We have used comparative genomics between a strain that releases Tcd and a strain that does not, together with a cosmid based *E. coli* heterologous expression system to investigate this hypothesis. Here we present evidence that toxin secretion is enhanced by a lipase (Pdl1), the activity of which is controlled by a small tightly linked antagonist protein (Orf53). Homologues of these genes can be seen to be linked to many virulence loci in other pathogens and we suggest that this work represents the first characterised example of an important and widespread secretion enhancement mechanism.

## Materials and Methods

### Cosmid identification and insertional mutagenesis

The c1AH10 was identified from a *Pl* W14 cosmid library [18] by aligning cosmid end sequences to the *tcd* pai sequence using SeqMan. The *E. coli* c1AH10 clone was tested for oral toxicity as described below. Insertional mutagenesis was performed with EZ::TN<TET1> transposon (Epicentre) to generate and characterise an insertion mutant sub-library as previously described [22]. Briefly, insertion mutant cosmid clone colonies were picked and DNA was prepared on a RoboPrep plasmid preparation robot (MWG Biotech). The insertion site of the transposon in each mutant was determined by sequencing out from the transposon with a transposon specific primer using an ABI3700 nucleotide sequencer (Applied Biosystems). Sequences were assembled onto the c1AH10 template sequence by using the LASERGENE software package (DNASTAR, Madison, WI) allowing the transposon insert sites to be determined.

### Cloning and recombinant expression of *pdl1* and flanking genes *orf54* and *orf53*

The *pdl1*, *orf54* and *orf53* genes were amplified from *P. luminescens* strain W14 genomic DNA using rTth DNA polymerase (Applied Biosystems). Polymerase chain reaction (PCR) conditions were 1.2–1.6 mM magnesium acetate, 2 mM each dNTP and 1 mM each primer. Thermocycling was performed as follows: 93°C for 30 s; 55°C for 30 s and 68°C for 3 min, for 30 cycles and a final 68°C incubation for 10 min. PCR primers, used for cloning into pET-28a(+), pCDF-1b (Novagen), and the arabinose inducible expression plasmid pBAD30 were designed to include unique restriction sites for subsequent cloning. The primer sequences (5′ to 3′) used for cloning the *pdl1*, *orf54* and *orf53* genes into pBAD30 were as follows:

- ***pdl1*** **only:** w14PDL-XbaIf: ATTcTAgaGGAAAGAGTATCAATGAGC;
  w14PDL-HindIII R: ATaagcttTCATACAGACAGTTCC;
- ***pdl1*** **and its upstream region:** Nco-5-P-pdl: CCccatggACTCCTTAGATGGTGTTAATCG;
  BamH-3-pdl: TCggatccTCATACAGACAGTTCCTG;
- ***pdl1*** **+** ***orf54*** **:** w14short-HindIIIR: ATaagcttTTACTTTATGTAACGGG;
- ***pdl1*** **+** ***orf54+orf53*** **:** w14long-HindIIIR: ATaagcttTACTTTATTTTCCAGTAG.

For His-tagged cloning, *pdl1* and *orf54* were initially cloned into pET-28a(+) as His-fusion proteins and then subsequently PCR amplified from this template as a His-fusion for cloning into pBAD30. The primer sequences (5′ to 3′) were as follows:

W14PDL-XbaIf: ATTcTAgaGGAAAGAGTATCAATGAGC;

28a-r-XhoI-pdl: ATATATCTCGAGTACAGACAGTTCCTGT;

30-r-SphI-PET28: ATATATGCATGCTAGTTATTGCTCAGCGG;

W14O53-XbaIF: ATTCTAGAATCTAAATGCCAACATGAG;

28a-r-XhoI-O53: ATCTCGAGCTTTATTTTCCAGTAGTC.

Following PCR, the products were purified (Millipore Montage PCR column as per instructions), cut with the appropriate restriction enzyme, and then re-purified prior to ligation and cloning. Plasmid DNA from pET-28a(+), pBAD30 and pCDF-1b expression vectors was prepared (Qiagen miniprep kit as per instructions) and co-digested with the relevant restriction enzymes. Ligations were performed at a 3∶1 molar excess of insert to vector using the Promega T4 DNA ligase rapid ligation system. Aliquots of the ligation reaction containing pET-28a(+) or pBAD30 vectors were electroporated into Epicentre Transformax EC100 *E. coli* and recovered on Luria–Broth (LB) agar containing 100 µg/ml ampicillin. Ligation containing pCDF-1b vector was transformed into chemically competent BL21 *E. coli* (Invitrogen) and recovered on LB agar containing 50 µg/ml Streptomycin. Correct constructs were selected by restriction digest of DNA prepared from candidate clones and verified by subsequent sequencing and stored at −80°C in 15% glycerol. Positive clones were subsequently induced for protein expression. Glycerol stocks were used to inoculate 5 ml of fresh LB media supplemented with 0.2% glucose (w/v) and the appropriate antibiotic for selection. Bacteria were grown overnight at 37°C with shaking, 1 ml of this culture was then harvested and resuspended in 100 ml of the same media and incubated at 37°C until an OD600 of 0.7–0.9 was achieved. Cells were then harvested at room temperature by centrifugation at 4000 rpm for 10 min. The pellet was re-suspended in 100 ml of fresh LB, supplemented with the appropriate antibiotic and 0.2% (w/v) of the pBAD30 promoter inducer L-arabinose or 1 mM IPTG for pCDF-1b construct. Cells or trichloroacetic acid (TCA) precipitated and concentrated supernatants from overnight induced cultures were collected for analysis using SDS-PAGE to confirm expression.

### Complementation assays

The c1AH10 derived cosmids containing transposon insertions into the *pdl1* gene (*pdl1* KO1-mutant) or in the pWEB vector backbone (CVI-wt) were isolated from the *E. coli* Pl W14 library clones and each transformed into chemically competent *E. coli* BL21 cells. To complement the mutant *pdl1* gene, *E. coli* BL21 cells were co-transformed with both the *pdl1* KO1-mutant cosmid and the pCDF-1b:*pdl1* construct. Transformant clones were recovered on LB agar containing the appropriate antibiotics. Positive clones containing both constructs were confirmed both by colony PCR and by DNA isolation and identification of cosmid and plasmid DNA using gel electrophoresis. Single colonies were subcultured in 5 ml of LB with the appropriate antibiotics and were grown overnight at 28°C with shaking. Larger subcultures were inoculated with these overnight pre-cultures (1/100 v/v dilution) and grown at 28°C with shaking for 72 h to be used in bioassays. Whole cultures, washed cells and supernatants were used in the *M. sexta* neonate feeding bioassays.

### Oral bioassay against *Manduca sexta* caterpillars

Supernatants or washed cells were diluted in 1× phosphate-buffered saline (PBS) and applied to 1 cm^3^ disks of artificial wheat germ diet as previously described [13]. Treated food blocks were allowed to dry for 30 min and two neonate *M. sexta* larvae were placed on each food block before incubation at 25°C for 7 days. Larvae were then scored for mortality and weighed. Larval growth differences are then expressed either as the direct mean values or as relative weight gain (RWG) means normalised to control groups [13]. The control groups are typically *E. coli* culture conditioned medium. RWG means are calculated using the following formula: RWG mean = 1+((sample mean−control mean)/control mean).

### Cell fractionation

*P. luminescens* W14 (*Pl* W14) was electroporated with various expression constructs using a standard *E. coli* optimised protocol. Transformants were confirmed by restriction digest of plasmid DNA prepared using a Qiagen kit as per manufacturer's protocol. *Pl* W14 carrying the *pdl1* or *orf53* expression constructs were induced with arabinose and cultured for different time points (3 h, 1 day and 2 days). Cells were washed in 1× PBS, normalized to an optical density of 10.0, and lysed by sonication (10 s on and 10 s off 45% power regime for 3 min). Unbroken cells were removed by low speed centrifugation. One millilitre of each cell-free lysate was fractionated into the soluble (cytoplasm and periplasm) and insoluble (inner and outer membrane) fractions by ultracentrifugation (28 000 r.p.m. for 2 h in a Beckman SW40Ti rotor). The insoluble fraction was resuspended in 1 ml of PBS to restore its original concentration relative to the soluble fraction proteins in the lysate. Supernatant samples were prepared and concentrated using TCA precipitation.

### Western blot

Protein sample amounts and quality were initially assessed by standard Coomassie blue staining of SDS-PAGE gels. For western blotting, protein fractions were separated by 1 dimensional SDS-PAGE and western blotted onto Nitrocellulose using a Biorad semi-dry blotter. We probed these membranes using a standard protocol with the following antibodies: TcdB1 (C terminal) anti-peptide antibody (raised against aa856- YSSSEEKPFSPPNDC-aa869), monoclonal anti-poly-histidine antibody (SIGMA), anti-β-Lactamase antibody (Millipore) and anti-SecG (a kind gift from Prof. Hajime Tokuda, University of Morioka). Immune-reactive bands were visualized using alkaline phosphatase-conjugated anti-rabbit or anti-mouse secondary antibody (SIGMA) at 1∶5000 and developed using NBT-BCIP agent. Anti-β-Lactamase western blots were performed to ensure no contamination of soluble material in the membrane fraction and anti-SecG western blots ensured no contamination of the membrane material in the soluble fraction.

### RT-PCR

Total RNA was extracted from normalised cell number samples (OD_600_ of 0.5) of *E. coli* or W14 cells at different time points using RNeasy kit (QIAGEN) according to the manufacturer's protocol. In all cases, the RNA was initially quality controlled by performing a standard Taq PCR reaction, using the intended primer pairs to ensure no contaminating DNA. RT-PCR was performed using One-step RT-PCR kit (QIAGEN). Thermocycling was performed as follows: 50°C for 30 min; 95°C for 15 min; 94°C for 30 s; 50°C for 30 s and 72°C for 1 min, for 25 cycles and a final 72°C incubation for 10 min. Primers, which were designed to include unique sequence of different target genes, were described as follows (5′-3′):

tccC5-F: CAGGCGGAACAGGTGATTAT; tccC5-R GAGTTGGATCTGCGGTCAAT;

tcdA1-F: TTGAGAGCGTCAATGTCCTG; tcdA1-R; TATCCGCGGCTCTGTCTAGT;

tcdB1-F: TGGAAGCCTCGATATCATCC; tcdB1-R: ATAGGCCAGTTCCAGTGGTG;

orf53-F: AAATTACGTCTGGATGTGAAG; orf53-R: CCAGTAGTCTATCGTTTGGCG;

pdl-F: GGGAACAATAAGCAGGGTGA; pdl-R: GGTGACGGCGATAACAACTT;

tccC2-F: ATCGGGGTGTTCTCAGTACG; tccC2-R: TTCTGTTTGGCTGTTTGCTG

### Haemolysis assays

**(i) Plate assay:** *E. coli* carrying pBAD30-*pdl1* were plated onto LB agar supplemented with ampicillin (100 µg/ml) and sheep red blood cells (SIGMA). For induction of *pdl1* expression 0.2% (w/v) arabinose was also included. Plates were incubated at 37°C overnight before visualisation. **(ii) Liquid assay:** These were performed as described elsewhere [23]. Briefly, washed cells were isolated from induced and un-induced pBAD30-*pdl1* cultures and sonicated. For induction, 0.2% (w/v) arabinose was added to cultures during exponential growth phase. Subsequently, 50 µl of red blood cells were suspended in phosphate buffered saline (PBS), added to 150 µl of either control buffers, induced or un-induced bacterial lysate before incubation at 37°C. After 24 hours, the whole red blood cells were removed by centrifugation at 5 rpm for 5 min, and the extent of haemoglobin release within the reaction supernatant was measured at an optical density of 540 nm in a spectrophotometer. Percentage haemolysis was calculated as [(OD_540_ treatment sample/OD_540_ total lysis)×100].

### Accession numbers

NCBI accession numbers for the proteins/genes described in this study are as follows: Pdl1, AAL18491; Orf53, AAL18489; TcdA1, AAL18486; TcdB1, AAL18487; TccC5, AAO17210; Orf54, AAL18490; Orf47, AAL18483 and Orf48, AAL18484.

## Results

### TC secretion by different strains of *Photorhabdus luminescens*

**Figure 2:**
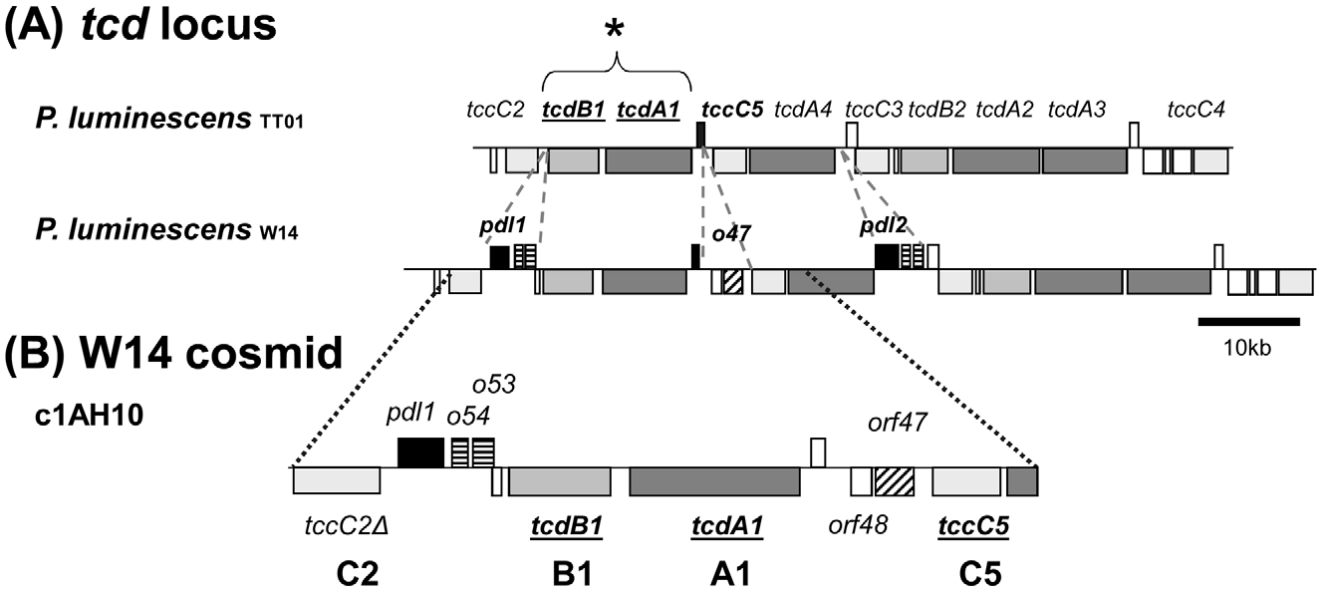
The *tcd* Pathogenicity islands of *P. luminescens*. (**A**) A comparison of the *tcd* pathogenicity islands (pai) of *P. luminescens* strains TT01 and W14. Note in the *tcd* pai, it is the *tcdA1* and *tcdB1* genes previously that have been associated with oral toxicity to *M. sexta* (see *). Several genes present in the *Pl* W14 *tcd* pai which are absent from *Pl* TT01 are indicated by the dotted lines. These include *pdl1* and *pdl2* (filled black) which are predicted lipases and *orf54* homologues (horizontal stripes) which have type II secretion leader peptides. The *orf47* predicted gene product (diagonal stripes) also has a predicted lipase domain and the downstream *orf48* protein also has a type II secretion leader peptide. This suggests analogous roles to *pdl1* and *orf47*. (**B**) A *Pl* W14 cosmid clone (c1AH10) that is sufficient to allow *E. coli* to produce both cell associated and freely secreted oral toxicity similar to *Pl* W14. This cosmid contains intact copies of all three *tc* homologues (subunits A+B+C) known to be necessary for producing full oral toxicity. Note the *tccC2* gene is N-terminally truncated and not functional on this clone.

We compared *M. sexta* oral toxicity data with a phylogenetic analysis of the *P. luminescens* species [24]–[25]. Members of the clade exemplified by strain *Pl* W14 all produce orally toxic supernatants and cells, while those in the clade which include strain *Pl* TT01 exhibit orally toxic cells but not supernatants (Fig. 1C). It is important to note here that we are talking about toxicity and not infection as *P. luminescens* cannot infect the model insect *M. sexta* via the oral route. Despite this, limited microarray analysis confirmed that all *P. luminescens* strains encode homologues of the oral toxins *tcdA1* and *tcdB1* genes [26], suggesting other lineage specific genetic factors might control the release of the orally toxic Tcd complex off the cell and into the surrounding medium. Previous work has identified the *tca* and *tcd* locus in *Pl* W14 as responsible for oral toxicity to *M. sexta* [4]. With the availability of the *Pl* TT01 genome sequence [27] and the sequences of several *tc* loci of *Pl* W14 [20] we have been able to directly compare the *tca* and *tcd* loci. The *tca* locus has undergone genetic degradation in *Pl* TT01 and so cannot contribute to oral toxicity. Conversely the *Pl* TT01 and *Pl* W14 *tcd* pathogenicity islands are well conserved, although the *Pl* W14 locus also contains a number of additional genes absent from *Pl* TT01 (Fig. 2A). We speculated that these genes are responsible for the release of Tcd into the surrounding supernatant in *Pl* W14, and without them, the majority of the Tcd complex remains associated with the cell surface in *Pl* TT01. Previous immuno-gold localisation studies using a Tca polyclonal antibody to probe thin sections of *Pl* W14 confirmed that TC cross-reactive antigens were indeed localised on the surface of the bacterial cells [19].

### A heterologous expression model to study Tcd export

To study Tcd export we developed a heterologous model for expression and secretion in *E. coli*. Previously we PCR screened a *Pl* W14 cosmid library for clones that encompassed the *tcdA1B1* genes [18] and tested them for the production of orally toxic supernatants. We confirmed that cosmid c1AH10 produced both supernatant and cell associated oral toxicity, consistent with the *Pl* W14 phenotype. This cosmid encodes intact copies of all three necessary subunit genes, A, B and C, (*tcdA1 tcdB1* and *tccC5*), required for full toxicity (Fig. 2B). Nevertheless the roles of other genes on this cosmid were not known. Two small *Pl* W14 specific islands are present on this cosmid. The first small island encodes a gene named *pdl1*, the predicted protein product of which contains putative lipase and protease domains, in addition to two small self-similar tightly linked ORFs (*orf53* and *orf54*) each with type II secretion signal peptides but no other recognisable domains. The second island contains the genes *orf47*, which also has a predicted esterase/lipase domain but is unrelated to *pdl*, and *orf48* which again has a putative signal peptide leader sequence but no other recognizable domains. To validate this cosmid as a suitable model for studying Tcd expression and secretion we compared the transcription and translation of several genes on c1AH10 in *E. coli* with those in the original *Pl* W14 strain across the growth curve using RT-PCR (Fig. S1) and western blot analysis (Fig. S2B). RT-PCR analysis revealed that the A, B and C-subunit gene transcripts are present at all growth phases, but decline by 3 days. A similar trend in transcriptional levels is seen for both the cosmid and the W14 strains supporting its relevance as a model system. We note a peak in *pdl1* mRNA at the late phase of growth (OD = 2, around 8 h for W14) especially in the cosmid clone which is consistent with the onset of toxin secretion.

We designed an anti-peptide antibody against the C-terminus of the B-subunit (TcdB1) and used this to track the translation and location of the Tcd complex. We do not see B-subunit translation in either strain *Pl* W14 or the cosmid clone until 24 hours (Fig. S2A), which correlates with the appearance of the oral toxicity phenotype. The failure of the antibody to cross react with supernatant proteins from a W14 *tcdAB* KO strain [4] confirms the specificity of this antibody for the TcdB1 subunit. When Tcd is produced by *Pl* W14 and the cosmid clone, it is present in the membrane and supernatant fractions (Fig. S2B). This correlates with whole-cell and supernatant associated oral toxicity of both the cosmid clone and *Pl* W14. Nevertheless, as previously seen, the overall levels of Tcd production are low [28].

### Pdl1 enhances Tcd mediated cell surface and supernatant toxicity

**Figure 3:**
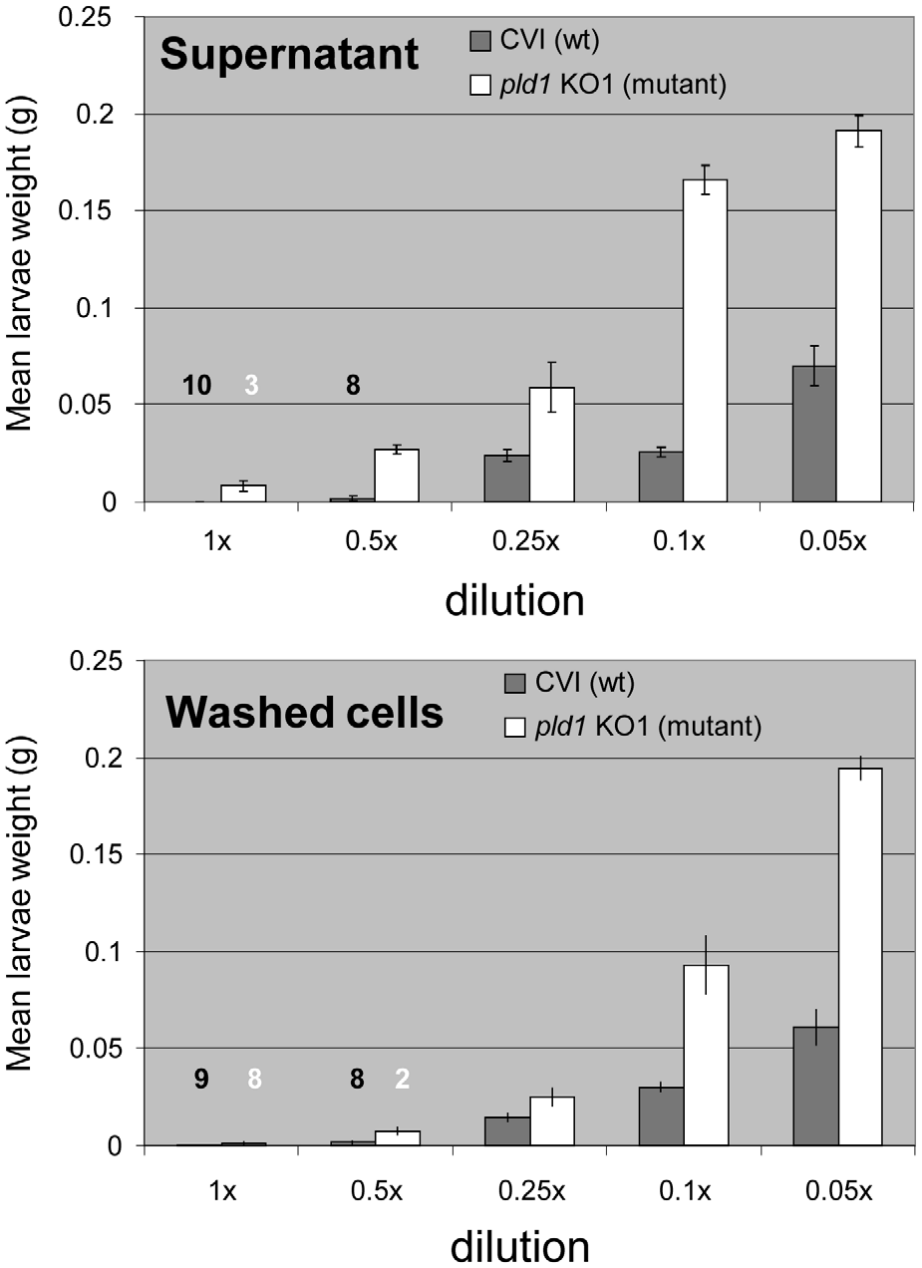
Insertion mutants in the c1AH10 *pdl1* gene are defective in Tcd supernatant release. Mean weight gain of cohorts of *M. sexta* neonates fed dilutions of washed cells or supernatants from 72 hour old cultures of *E. coli* containing the c1AH10 cosmid with transposon inserts in either the *pdl1* gene (*pdl1* KO1-mutant), white, or the pWEB cosmid vector backbone insertion (CVI-wt), shaded. Error bars represent the standard error. The more potent the toxic effect, the smaller the mean larval weight. Note the loss of toxicity at higher dilutions of the supernatants of the *pdl1* knock out cosmid strain. The figures in white and black above the bars represent larval mortality numbers during the 7 day assay.

An *in vitro* transposon mutagenesis kit was used to create a library of transposon mutants of cosmid c1AH10. Insertion knock-out mutants in all genes were identified by sequencing out from the transposon into the flanking cosmid sequence. The oral toxicity of cells and supernatants of this panel of cosmid mutants (in *E. coli*) was then examined by *M. sexta* oral bioassay. As expected this confirmed that all the three subunit genes were required for toxicity in *E. coli* (at least under these “native” expression levels). In addition however we can see that insertion mutagenesis of *pdl1* significantly lowered toxicity of the supernatant and to some extent the cells (Fig. 3). A panel of transposon mutants was transformed into *Pl* TT01 which is normally unable to release its native Tcd into the surrounding supernatant. Oral bioassay revealed that a cosmid in which the transposon was inserted into the cosmid vector backbone did indeed generate orally toxic supernatants (Fig. S3) as it does in *E. coli*. Importantly the *pdl1* cosmid mutant in the *Pl* TT01 background was again unable to produce orally toxic supernatants. Furthermore insertion into either of the *Pl* W14 C- or B-subunit genes also failed to produce toxic supernatants, suggesting they were essential to the Pdl1 mediated Tcd export enhancement. Interestingly, cosmid clones in which the A-subunit gene (*tcdA1*) was interrupted were still able to produce orally toxic supernatants. This indicated that a *Pl* TT01 A-subunit homologue was able to trans-complement for the cosmid *Pl* W14 equivalent. Conversely, the inability of the *Pl* TT01 B- and C-subunit homologues to trans-complement the equivalent cosmid mutants revealed that the *Pl* W14 Pdl1 does show some substrate specificity. As further confirmation of this we cloned *pdl1* alone into the arabinose inducible expression plasmid pBAD30 for over expression in *Pl* TT01. Consistent with our hypothesis, this construct did not significantly increase oral toxicity of the supernatants (data not shown).

### Pdl1 increases Tcd export into the supernatant

**Figure 4:**
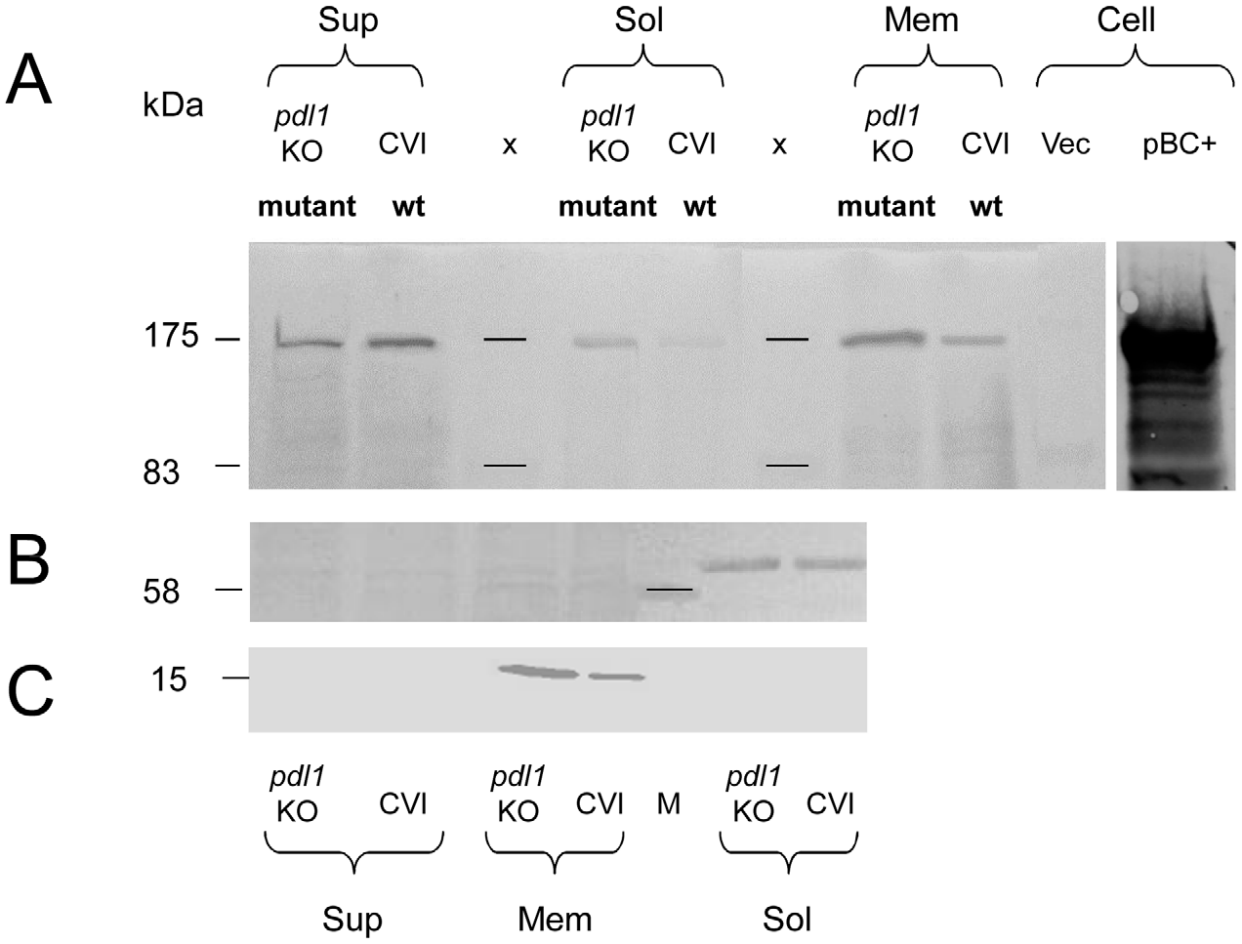
Pdl1 increases TcdB release into the supernatant. (**A**) Western blot analysis of culture supernatants (sup), and cellular membrane (mem) and soluble fractions, which includes cell cytosol and periplasm fractions (sol). Preparations from 24 h cultures of *E. coli* containing c1AH10 with transposon insertions into either the pWEB vector backbone (CVI-wt) or the *pdl1* gene (*pdl1* KO-mutant). X = marker and Vec = whole cell pWEB negative vector control. The presence or absence of Tcd is visualised using the antibody raised against the C-terminus of the B-subunit. Protein from an induced clone containing a B and C-subunit expression construct (pBC+) is included as a positive control. (**B**) An anti-β-Lactamase western blot was performed to ensure no contamination of soluble material in the membrane fraction. (**C**) An anti-SecG western blot was performed to ensure no contamination of membrane material in the soluble fraction.

In order to understand the effect of Pdl1 on Tcd export we examined cell soluble and membrane fractions and culture supernatants for Tcd by western blotting using an anti B-subunit antibody. We compared these fractions from *E. coli* harbouring the wild-type (Fig. 4 - CVI) and *pdl1* mutant (Fig. 4 – *pdl1* KO) cosmids (Fig. 4). The wild-type cosmid strain secretes Tcd into the supernatant with very little remaining associated with the soluble fraction, which consists of cytoplasmic and periplasmic fractions. Conversely, the *pdl1* mutant cosmid showed a reduction in the overall amount of Tcd secreted into the supernatant but an increase in amounts in the soluble and membrane fractions. This is despite a reduction in cell associated toxicity of the mutant (Fig. 3), suggesting that Pdl1 increases the efficiency of the normal secretion process. The soluble fraction shows the Tcd is accumulating in either in the cytoplasm and/or periplasm while the membrane fraction shows at least some is accumulating in either the inner or outer membranes. The role of Pdl1 in increasing overall export and release of Tcd was supported with a complementation assay in *E. coli*. We co-transformed *E. coli* BL21 with both the *pdl1* knock out mutant cosmid and a *pdl1* expression construct, pCDF-1b:*pdl1*. We confirmed Pdl1 expression in these strains using SDS-PAGE and confirmed that trans-complementation restored supernatant toxicity close to levels seen in the “wild-type” parental cosmid strain encoding an intact copy of the *pdl1* gene (Fig. S4). Expression of Pdl1 from pCDF-1b:*pdl* in the absence of the *tcd* cosmid showed no toxicity as expected. These experiments confirmed that the transposon insertion in the *pdl1* KO1-mutant cosmid was affecting the *pdl1* gene only. Interestingly we also saw a slight increase in cell associated toxicity in this trans-complemented strain, consistent with the cosmid mutation studies. This indicates that Pdl1 not only increases Tcd release into the surroundings but also enhances overall expression levels.

### Activity and localisation of Pdl1 and Orf53

**Figure 5:**
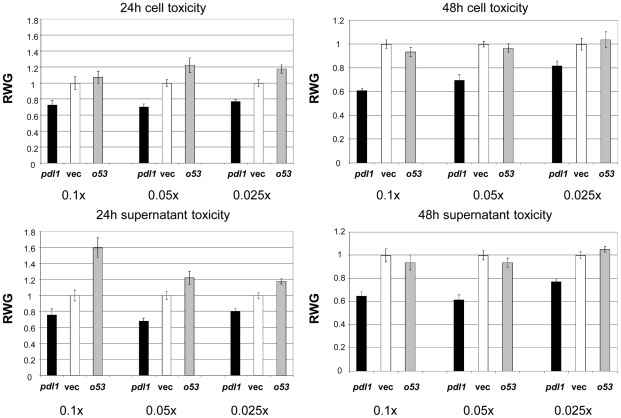
Pdl1 and Orf53 expression in strain W14 increases and decreases Tc toxin release respectively. *M. sexta* oral bioassay of dilutions (0.1×, 0.05× and 0.025×) of cells and supernatants from *P. luminescens* W14 cultures over-expressing C-terminally his-tagged Pdl1 (*pdl1*) or Orf53 (*o53*) from the pBAD30 expression vector. *Pl* W14 harbouring the induced empty pBAD30 expression vector (Vec) provides a control against which the data has been normalised. Bioassays were conducted using samples taken at 24 and 48 hours post induction and the mean Relative Weight Gain (RWG) of larvae is shown, in relation to the negative controls (Vec) which therefore give a RWG of 1. Note these are the same cultures examined by western blot in Fig. S5. Each data point is derived from the mean weight of 10 neonate larvae after seven days consumption. RWG-standard error bars are also shown.

C–terminal His tagged fusions of *pdl1* and the adjacent *orf53* were cloned into the arabinose inducible pBAD30 expression plasmid (*orf54* was not used as it appears to be translationally coupled to *pdl1*). These constructs were transformed into *Pl* W14, induced for 3, 24 and 48 hours and supernatant, membrane and soluble cell fractions prepared. The location of the His-tagged Pdl1 and Orf53 proteins were examined using western blotting with an anti-his tag antibody. Pdl1 was found in the soluble and membrane fractions for up to 2 days, but was always absent from the supernatants (Fig. S5A). Interestingly, the Orf53 protein was seen in the soluble and the membrane fractions at 3 h. The full length, unprocessed protein is seen in the soluble fraction while the majority of protein in the membrane fraction is a slightly shorter processed form, presumably with the type II signal leader removed (Fig. S5B). By 24 h the protein levels have fallen and are absent by 48 h. The oral toxicity of cells and supernatants from these same samples was tested (Fig. 5). At 24 h, over-expression of *pdl1* in *Pl* W14 increases the level of oral toxicity of both cells and supernatants relative the vector control. Conversely over-expression of *orf53* lowers toxicity of the supernatant. Interestingly by 48 h the *orf53* over-expression strain returns to the normal wild-type level of oral toxicity. This correlates to the disappearance of the Orf53 protein on the western blots by two days. Pdl1 over-expression continues to increase oral toxicity even at 48 h. This supports a model whereby Pdl1 increases Tcd export and Orf53 acts antagonistically to inhibit this effect.

### Investigating the mode of action of Pdl1

**Figure 6:**
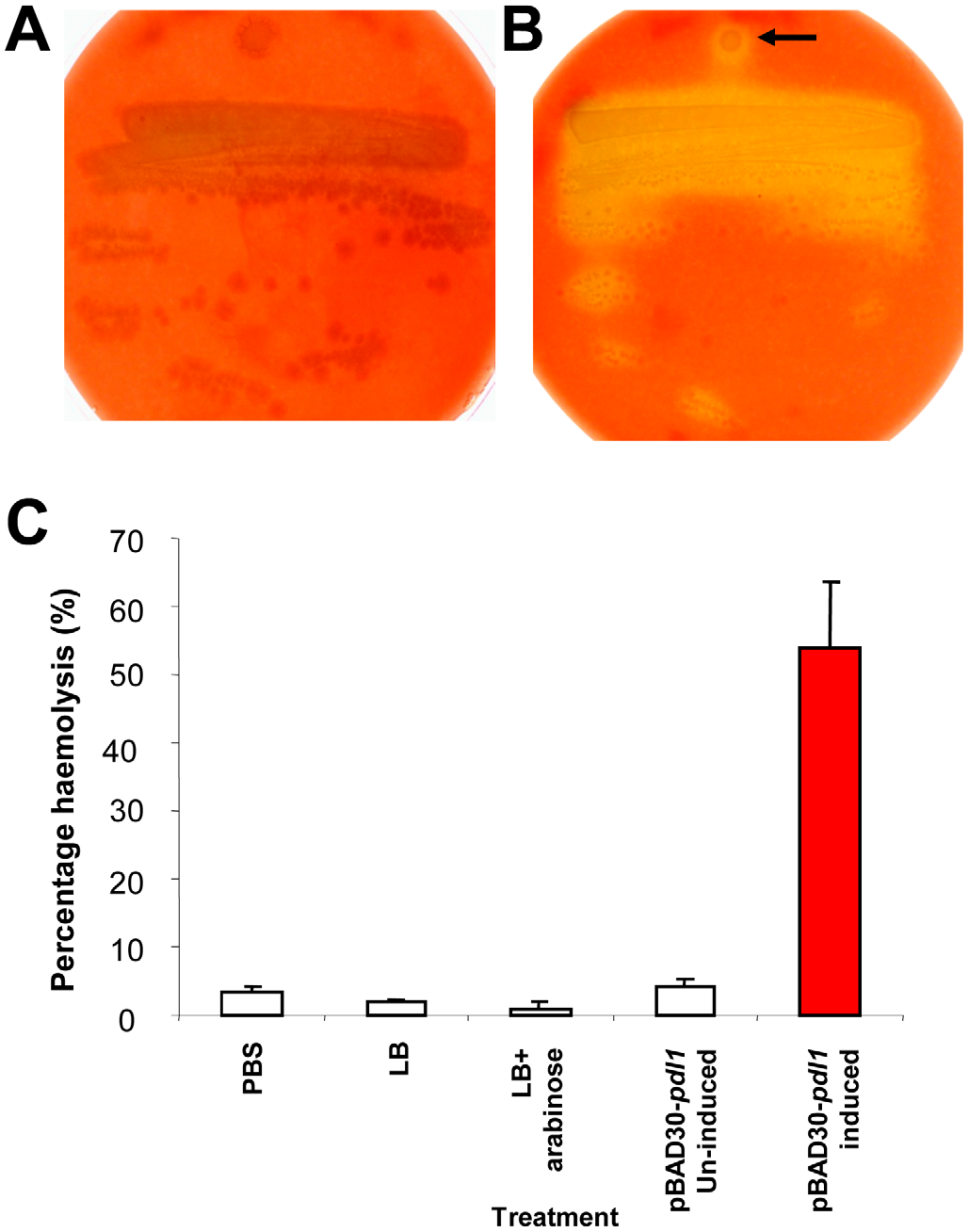
Pdl1 exhibits some haemolytic activity. A weak haemolytic phenotype displayed by the *E. coli* heterologous pBAD30-*pdl1* based expression strain. The un-induced (**A**) and arabinose induced (**B**) expression strain on agar plates containing sheep red blood cells. Note the diffuse zone of haemolysis surrounding the induced *E. coli* expressing Pdl1 (arrow). (**C**) Haemolysis of sheep red blood cells by heterologously expressed Pdl1 in a liquid assay. Controls include, PBS alone, LB medium alone, LB supplemented with 0.2% w/v arabinose and the un-induced pBAD30-*pdl1* expression strain. Results are expressed as a percentage haemolysis, where 100% corresponds to the amount of haemoglobin released by complete lysis of the red blood cells using water.

The demonstration that Pdl1 is not found in the supernatant fraction of *Pl* W14 cultures confirms that the increase in Tcd dependant supernatant toxicity is not the result of a continued physical association of Pdl1 with the secreted Tcd complex (Fig. S5). Protein alignments indicate that Pdl1 and Pdl2 contain putative protease and lipase like catalytic domains (Fig. S6). We therefore tested the possibility that Pdl1 might directly modify the Tcd complex in order to increase its specific activity. We mixed Tcd containing samples with lysate from the induced *E. coli* pBAD30-*pdl1* expression strain and performed oral toxicity bioassays (Fig. S7). These experiments confirmed that Pdl1 had no effect on the activity of Tcd isolated from any of the cell compartments, suggesting it is not likely to proteolytically “activate” the complex. In order to test if Pdl1 has any measurable lipase activity we tested its ability to lyse sheep red blood cells *in vitro*. We did this using both standard blood-agar haemolysis plates and a liquid assay based on measuring haemoglobin release. Pdl1 was heterologously expressed using the *E. coli* pBAD30-*pdl1* expression construct. To ensure any observed lysis was not due to the *E. coli sheA* dependant cryptic haemolytic activity [29], the pBAD30-*pdl1* construct was first electroporated into the *E. coli sheA* mutant CFP201. Fig. 6 confirms that after overnight incubation, Pdl1 expressing cells show limited lysis of sheep erythrocytes consistent with lipase activity. Finally we investigated the effect of *pdl1* over-expression and the co-expression of the tightly linked *orf54* and *orf53* upon *E. coli*. Pdl1 expression induced the release of specific protein species into the culture supernatant when compared to a vector only *E. coli* control (Fig. S8). Interestingly the most abundant of polypeptide appears to be a fragment derived from a RhsA-like protein of *E. coli* as well as other predicted membrane bound proteins such as the transporter MchF and the outer membrane lipoprotein PgaB. Interestingly when *orf54* was co-expressed with *pdl1*, the abundance of the various released polypeptides decreased, and was in most cases abolished when both *orf54* and *orf53* were both co-expressed. This further supports our findings with Tcd secretion that Pdl1 is able to influence protein export and that the tightly linked *orf54* and *orf53* gene products serve to repress the function of Pdl1 in a gene dose dependant manner.

## Discussion

Transcript and Western blot analysis revealed that the TC subunits are under post-transcriptional regulation, and that their translation is timed with secretion and release from the cell. The TC toxin subunits encode no recognised export signal sequences and show no similarity to either two partner secretion proteins, or known auto-transported proteins. Nevertheless these large proteins are secreted from the cell and assembled into a large complex without the need for cell lysis as confirmed by the viability of the *E. coli* cosmid clones. This suggests that either the TC subunits are auto-transported or that they may be secreted by a chromosomally encoded system conserved in both *Photorhabdus* and the *E. coli* laboratory strains used. The mechanism of TC subunit secretion is currently under investigation in our laboratory however it is clear that the exported TC becomes localised to the membrane fraction, and is accessible to the outside of the cell, potentially located on the outer membrane. The B and C subunit proteins can both be seen to contain tyrosine-aspartic acid (YD) repeat motifs which in other proteins are involved in binding carbohydrates [30]. It is possible that the YD repeats found in the B- and C-subunits are responsible for the wall association of the TC with membrane or extracellular polysaccharide components. Another possibility is that one or more of the subunits is a lipoprotein and is attached by a lipid anchor, although they do not exhibit the usual lipidation export signal peptide. Bacterial precursor lipoproteins can be translocated across the cytoplasmic membrane by either the Sec (general secretory) or Tat (twin arginine transport) pathways [31] and then lipid modified by a lipoprotein diacylglycerol transferase on a conserved cysteine. This cysteine is located in what is known as a lipobox motif at the end of the signal peptide [32]. The TC subunits contain no such leader peptides.

Our findings show that the Pdl1 protein can enhance Tcd secretion and that products of the small tightly linked genes are able to repress this effect. Our experiments confirmed that both Pdl1 and Orf53 are located in the membrane fraction although the exact location is not clear. Unfortunately we were unable to prepare isolated inner and outer membrane fractions from *Photorhabdus* in order to precisely localise the tagged Pdl1 and Orf53 proteins. It should be noted that similar problems were encountered in experiments with the closely related bacteria *Xenorhabdus nematophila* [33]. The presence of a type II Sec-dependant secretion leader on Orf53 does suggest that it can at least reach the periplasmic space, and indeed our experiments show both intact and processed forms consistent with cleavage of the signal peptide. PSORT analysis of the protein sequence gives a strong prediction for its localisation in the periplasmic space (certainty value = 0.930).

Results presented here suggest that the Pdl1 protein does not associate with or modify the Toxin Complex directly in order to increase its specific activity. Rather our data are consistent with a role in the enhancement of secretion. The over-expression of Pdl1 in *Pl* W14 increases the overall levels of both supernatant and cell contact dependant toxicity. One possible model infers that translation is coupled to export, with Pdl1 increasing the efficiency of secretion, leading to an overall increase in TC production. In this model, as cell surface binding sites become limited, then excess toxin is shed into the surrounding medium. Pdl1 may interact with the Tcd secretion mechanism on the cytoplasmic membrane during export. In this model, the Orf53 molecules would antagonise the activity of Pdl1 by interactions on the periplasmic face of the inner membrane. While we confirm lipase activity of Pdl1, the predicted presence of a serine protease catalytic motif does suggest that while proteolytic activity may not be involved in “activation” of the TC, it could still play a role in enhancing secretion.

Experiments in which cosmid mutants were transformed into strain TT01 suggest that the Pdl1 mediated release is dependent upon the BC sub-complex and is independent of the A-subunit. It should be noted that C-subunits belong to a much larger family that includes the Recombination Hot Spot genes (Rhs) of *E. coli*. These genes have an unusual structure with conserved N-termini, a highly conserved central core region and variable C-terminal “tails” [30], [34]. In the case of the C-subunit proteins we know the variable C-terminal domains encode toxic effectors [9]. The N-terminal domain of RhsA has been shown to be involved in the biosynthesis and export of capsular polysaccharide in *E. coli* although the exact mechanism remains cryptic [35]. Interestingly, Rhs-core like genes are often tightly linked to *vgrG* genes which in some cases have been shown to be contact dependant cytotoxins exported via type VI secretion machinery, temping the speculation of an equivalent toxin secretion and anchoring role [36].

An examination of the full genome sequence of *P. luminescens* TT01 shows only a single *pdl* homologue unlinked to any *tc* genes. Interestingly this is however closely linked to a putative type III effector toxin gene, *plu3163* (HopT1-2 homologue). The emerging human pathogen *P. asymbiotica* can however be seen to contain several genomic islands (GIs) containing repeats of *pdl-orf54* like genes. When cloned on cosmids, three of these *pdl*-GIs have been shown to be toxic to insects and nematodes [37]. Insertional mutagenesis of a cosmid containing the *pdl*-GI_2 region (Fig. S9A) confirmed that knock-out of either the putative toxin gene (a *vgrG* homologue) or two of the *pdl* homologues significantly reduced toxicity of the bacterial cultures [37]. When compared to genomic sequences in the public databases *pdl*-like genes may be seen in other pathogenic bacterial genera including *Vibrio* and *Pseudomonas* [38]–[39]. In many genomes we can see tight linkage between type VI secretion operons and *pdl*-*orf54* homologue gene pairs, suggesting they also play a role in these secretion systems (Fig. S9B). Taken together, these findings suggest that Pdl homologues may represent a novel general mechanism for enhancing secretion of toxins from Gram-negative bacteria.

## Supporting Information

Figure S1 **Cosmid gene transcription is similar to that in the parent strain** ***Pl*** **W14.** Comparisons of the transcription of genes present on c1AH10 in *E. coli* and in the original strain *P. luminescens* W14. RT-PCR amplification from total RNA prepared from equivalent cell numbers at different points in the growth curve *in vitro* at 30°C shaking in LB medium. Note the functional Tcd subunit genes are in dotted boxes and the *pdl1* is boxed in solid outline. The key to the gene location on the cosmid is shown above. RNA samples were taken at early exponential (OD_600_ = 0.2, c.a. 2 h), late exponential (OD_600_ = 1.0, c.a. 5 h), stationary phase (OD_600_ = 2.0, c.a. 8 h) and 72 hours into growth respectively. The presence of oral toxicity in the supernatants is indicated, with (−) meaning no toxicity and a range of toxicity from partial (+) to maximal (++++). The star indicates the expression of *pdl1* which peaks at the time of TC release into the supernatant.
(TIF)

Figure S2 **TcdB1 expression in** ***Pl*** **W14 and the cosmid clone.** Expression was tracked using an anti-peptide raised against a peptide located in the C-terminal region of the B-subunit TcdB1 (aa856-YSSSEEKPFSPPNDC-aa869). (**A**) A qualitative comparison of culture supernatants of wild type *Pl* W14 and a strain in which *tcdA* and *tcdB* have been deleted (D-). The absence of cross reactivity in the *tcdAB* KO strain samples confirmed that the anti-peptide antibody used is specific to the TcdB1 B-subunit. The pBC+ lane represents a positive control of whole cells of an induced *E. coli* pBAD30 based heterologous *tcdB1-tccC1* expression strain. (**B**) A qualitative comparison of the sub-cellular location of TcdB1 in *Pl* W14 and *E. coli* containing c1AH10 (cos). Sup = supernatants; Sol = cytoplasmic+periplasmic fractions; Mem = membrane fractions. Samples were prepared from cultures generating orally toxic supernatants after 72 hrs growth *in vitro* at 30°C (* indicates confirmation of oral toxicity by bioassay).
(TIF)

Figure S3 **Oral toxicity of cosmid transposon mutants in** ***Pl*** **TT01.** (**A**) Map of cosmid c1AH10 showing transposon insertion points tested for supernatant oral toxicity when transformed into *P. luminescens* TT01. Filled inverted triangles represent transposon insertion points that maintained toxicity (T = toxic), while those which abolished toxicity are shown as open triangles (NT = not toxic). (**B**) Mean relative weight gain (RWG) of cohorts of *M. sexta* neonates fed supernatants from 72 hrs cultures of *Pl* TT01 containing the various cosmid mutants. Note the data has been normalised to that from the TT01 strain containing the c1AH10 with a transposon insertion into the pWEB vector backbone (CVI) which produced the maximum toxicity. Error bars represent the standard error. Note the black bars show a failure of the *pdl1* knock out mutants to secrete active toxin and the hatched bars indicate that both the B and C-subunit (*tcdB1* and *tccC5*) genes are also required for release of toxin. Insertion into the A-subunit gene (*tcdA1*) remained fully toxic, so must be able to be trans-complemented by chromosomal A-subunit homologues.
(TIF)

Figure S4 **Trans-complementation of the** ***pdl*** **-knock out cosmid strain restores the Tcd release phenotype.** Mean weight gain of cohorts of *M. sexta* neonates (n = 12) fed with whole cultures, supernatants or cells from 72 hour old 28°C grown cultures of *E. coli* containing the CVI-wt cosmid (with transposon inserts in the pWEB backbone), the *pdl1* KO1-mutant cosmid (with transposon inserts in the *pdl1* gene), both the *pdl1* KO1-mutant cosmid and the pCDF-1b:*pdl1* vector (expressing Pdl1), and the pCDF-1b:pdl1 vector alone. Error bars represent the standard error. The more potent the toxic effect, the smaller the mean larval weight. Note the restoration of toxic activity in the supernatants *pdl*1 KO1-mutant strain expressing trans-complemented Pdl1.
(TIF)

Figure S5 **In** ***Pl*** **W14, Pdl1 and Orf53 are not released into the supernatant.** Western blots of membrane (m), soluble cytoplasm+periplasm (sol) and supernatant (s) fractions from *Pl* W14 over-expressing C-terminally his-tagged Pdl1 (**A**) and Orf53 (**B**) from the arabinose inducible pBAD30 expression vector. Samples were taken at 3, 24 and 48 hours and continued arabinose induction was maintained throughout the incubation period. The native Shine-Dalgarno sequences are included in these constructs. The two arrows (**B**) indicate the presumed processed and full length forms of Orf53. Size markers are also shown (x). An anti-β-lactamase western blot (Anti-BLA) was performed as a control for loading amounts and the quality of the fractionation for both expression constructs.
(TIF)

Figure S6 **Pdl has potential protease and lipase domains.** Alignment of the predicted amino acid sequences of *Pl* W14 Pdl1 and Pdl2 (Genbank AY144119), with predicted products of the *A. oryzae mdlB* gene (Genbank D85895) and a *V. cholerae* hypothetical open reading frame, VC1418 (Genbank AE004220). The presence of the presumptive serine protease-like catalytic triad (S, D and H) is highlighted (red) alongside the conserved pentapeptide GHSXG (yellow) common to lipases and lipoprotein lipases.
(TIF)

Figure S7 **Pdl1 has no direct effect on the activity of Tcd.** Mean weight gain of cohorts of *M. sexta* neonates (n = 10) fed different Tcd containing cell fractions (soluble, washed whole cells or supernatants) which had been pre-incubated for 1 h at 28°C with sonicated cell extracts from either an induced *E. coli* pBAD30-*pdl1* expression construct (Pdl1) or an *E. coli* pBAD30 negative control (pBAD30). The Tcd fractions were isolated from the *E. coli pdl1* knock out cosmid strain (*pdl1*-KO). We also used *E. coli* pWEB fractions as a further negative control (pWEB). Standard error bars are shown. The more potent the toxic effect, the smaller the mean larval weight. Note the presence of added Pdl1 does not increase toxic activity of Tcd from any fraction. Data from a 7 day assay.
(TIF)

Figure S8 **The effects of Pdl1 and Orf53 over-expression on native protein release in** ***E. coli*** **.** The effect of *pdl1*, *pdl1+orf54* and *pdl+orf54+orf53* pBAD30 expression constructs on supernatant proteins released by the recombinant *E. coli*. All genes have their native Shine-Dalgarno sequences. Size markers are also shown (x). Note Pdl1 induces the release of several specific protein species (boxed). The inclusion of *orf54* and *orf53* (which are homologues of one another) reduce this effect in an additive manner. Putative MALDI-ToF identification of several of these *E. coli* protein species are indicated.
(TIF)

Figure S9 **Pdl homologues are associated with other toxin secretion genes in diverse bacteria.** (**A**) A *pdl-orf54* island of *P. asymbiotica* ^ATCC43949^ identified using RVA screening exhibiting insect toxicity on injection. Colour coding identifies homologous genes. Genbank locus tag numbers are given. The *pdl* and *vgrG* homologues were shown to be responsible for the toxicity of the *Pa pdl*-GI_2 virulence island were mapped by transposon mutagenesis (red inverted triangles) (**B**) *pdl-orf54* homologues are often tightly linked to other toxin secretion systems in diverse pathogens such as type VI secretion systems in *Vibrio* and *Pseudomonas*.
(TIF)
