# Supplementary figures and images for: Pdl1 Is a Putative Lipase that Enhances Photorhabdus Toxin Complex Secretion
Source: PLoS Pathog. 2012 May 17;8(5):e1002692. doi: 10.1371/journal.ppat.1002692 (PMC3355079; doi:10.1371/journal.ppat.1002692)

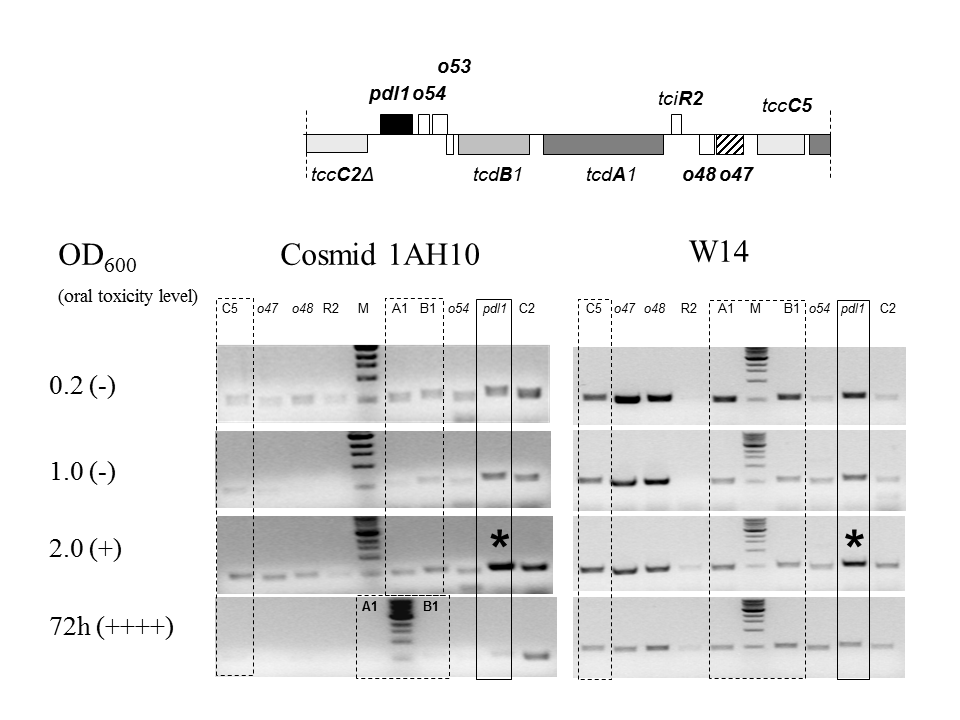

Supplement: Figure S1 — Cosmid gene transcription is similar to that in the parent strain Pl W14. Comparisons of the transcription of genes present on c1AH10 in E. coli and in the original strain P. luminescens W14. RT-PCR amplification from total RNA prepared from equivalent cell numbers at different points in the growth curve in vitro at 30°C shaking in LB medium. Note the functional Tcd subunit genes are in dotted boxes and the pdl1 is boxed in solid outline. The key to the gene location on the cosmid is shown above. RNA samples were taken at early exponential (OD600 = 0.2, c.a. 2 h), late exponential (OD600 = 1.0, c.a. 5 h), stationary phase (OD600 = 2.0, c.a. 8 h) and 72 hours into growth respectively. The presence of oral toxicity in the supernatants is indicated, with (−) meaning no toxicity and a range of toxicity from partial (+) to maximal (++++). The star indicates the expression of pdl1 which peaks at the time of TC release into the supernatant. (TIF) [file ppat.1002692.s001.tif]

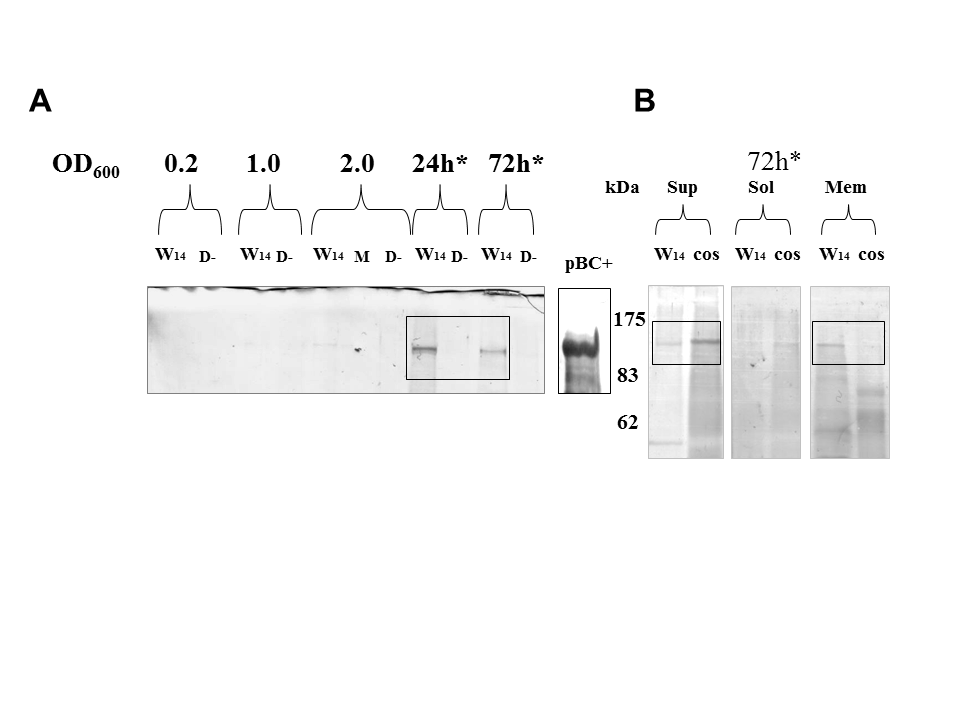

Supplement: Figure S2 — TcdB1 expression in Pl W14 and the cosmid clone. Expression was tracked using an anti-peptide raised against a peptide located in the C-terminal region of the B-subunit TcdB1 (aa856-YSSSEEKPFSPPNDC-aa869). (A) A qualitative comparison of culture supernatants of wild type Pl W14 and a strain in which tcdA and tcdB have been deleted (D-). The absence of cross reactivity in the tcdAB KO strain samples confirmed that the anti-peptide antibody used is specific to the TcdB1 B-subunit. The pBC+ lane represents a positive control of whole cells of an induced E. coli pBAD30 based heterologous tcdB1-tccC1 expression strain. (B) A qualitative comparison of the sub-cellular location of TcdB1 in Pl W14 and E. coli containing c1AH10 (cos). Sup = supernatants; Sol = cytoplasmic+periplasmic fractions; Mem = membrane fractions. Samples were prepared from cultures generating orally toxic supernatants after 72 hrs growth in vitro at 30°C (* indicates confirmation of oral toxicity by bioassay). (TIF) [file ppat.1002692.s002.tif]

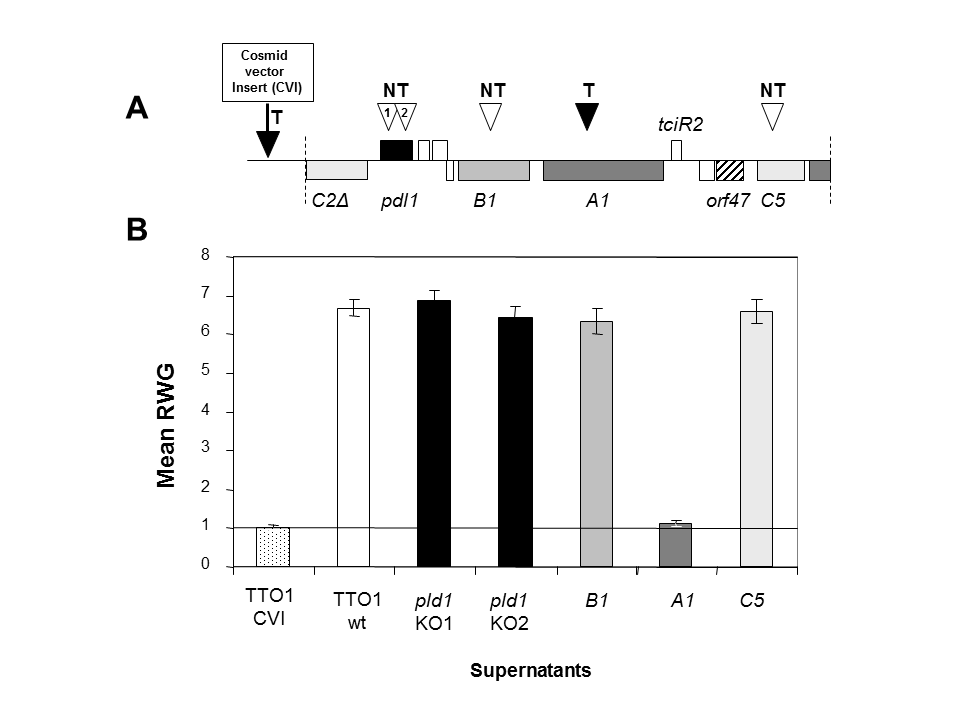

Supplement: Figure S3 — Oral toxicity of cosmid transposon mutants in Pl TT01. (A) Map of cosmid c1AH10 showing transposon insertion points tested for supernatant oral toxicity when transformed into P. luminescens TT01. Filled inverted triangles represent transposon insertion points that maintained toxicity (T = toxic), while those which abolished toxicity are shown as open triangles (NT = not toxic). (B) Mean relative weight gain (RWG) of cohorts of M. sexta neonates fed supernatants from 72 hrs cultures of Pl TT01 containing the various cosmid mutants. Note the data has been normalised to that from the TT01 strain containing the c1AH10 with a transposon insertion into the pWEB vector backbone (CVI) which produced the maximum toxicity. Error bars represent the standard error. Note the black bars show a failure of the pdl1 knock out mutants to secrete active toxin and the hatched bars indicate that both the B and C-subunit (tcdB1 and tccC5) genes are also required for release of toxin. Insertion into the A-subunit gene (tcdA1) remained fully toxic, so must be able to be trans-complemented by chromosomal A-subunit homologues. (TIF) [file ppat.1002692.s003.tif]

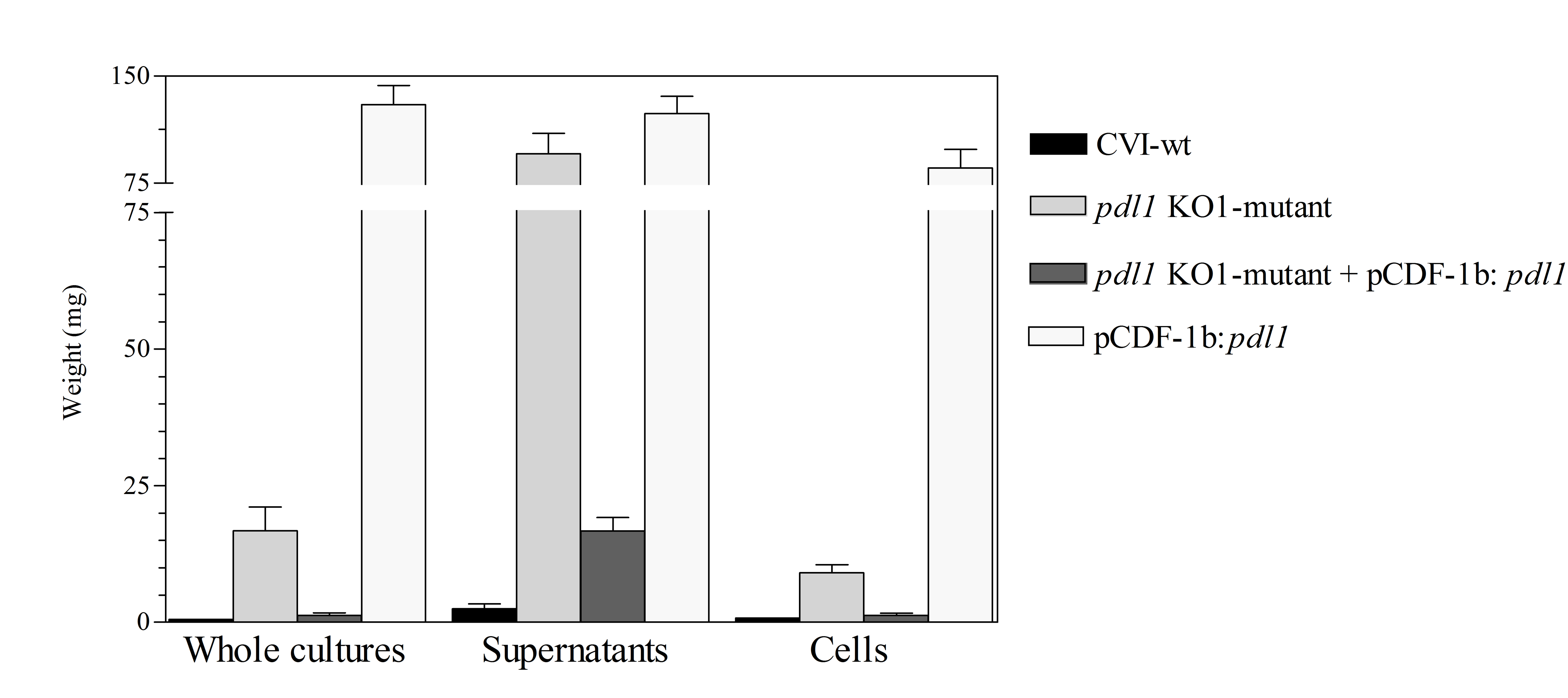

Supplement: Figure S4 — Trans-complementation of the pdl -knock out cosmid strain restores the Tcd release phenotype. Mean weight gain of cohorts of M. sexta neonates (n = 12) fed with whole cultures, supernatants or cells from 72 hour old 28°C grown cultures of E. coli containing the CVI-wt cosmid (with transposon inserts in the pWEB backbone), the pdl1 KO1-mutant cosmid (with transposon inserts in the pdl1 gene), both the pdl1 KO1-mutant cosmid and the pCDF-1b:pdl1 vector (expressing Pdl1), and the pCDF-1b:pdl1 vector alone. Error bars represent the standard error. The more potent the toxic effect, the smaller the mean larval weight. Note the restoration of toxic activity in the supernatants pdl1 KO1-mutant strain expressing trans-complemented Pdl1. (TIF) [file ppat.1002692.s004.tif]

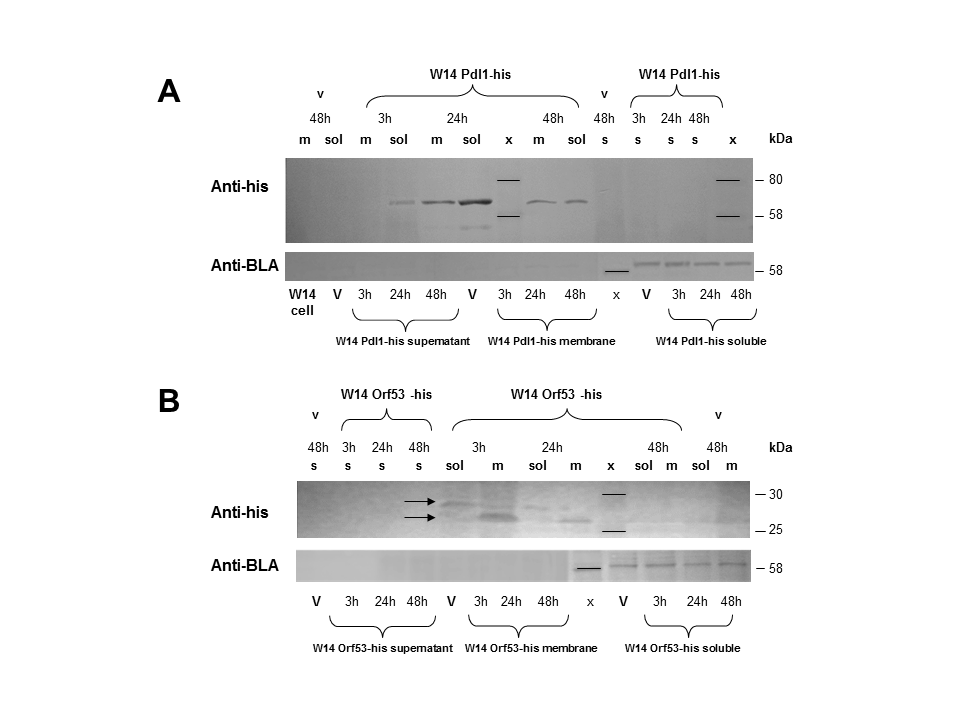

Supplement: Figure S5 — In Pl W14, Pdl1 and Orf53 are not released into the supernatant. Western blots of membrane (m), soluble cytoplasm+periplasm (sol) and supernatant (s) fractions from Pl W14 over-expressing C-terminally his-tagged Pdl1 (A) and Orf53 (B) from the arabinose inducible pBAD30 expression vector. Samples were taken at 3, 24 and 48 hours and continued arabinose induction was maintained throughout the incubation period. The native Shine-Dalgarno sequences are included in these constructs. The two arrows (B) indicate the presumed processed and full length forms of Orf53. Size markers are also shown (x). An anti-β-lactamase western blot (Anti-BLA) was performed as a control for loading amounts and the quality of the fractionation for both expression constructs. (TIF) [file ppat.1002692.s005.tif]

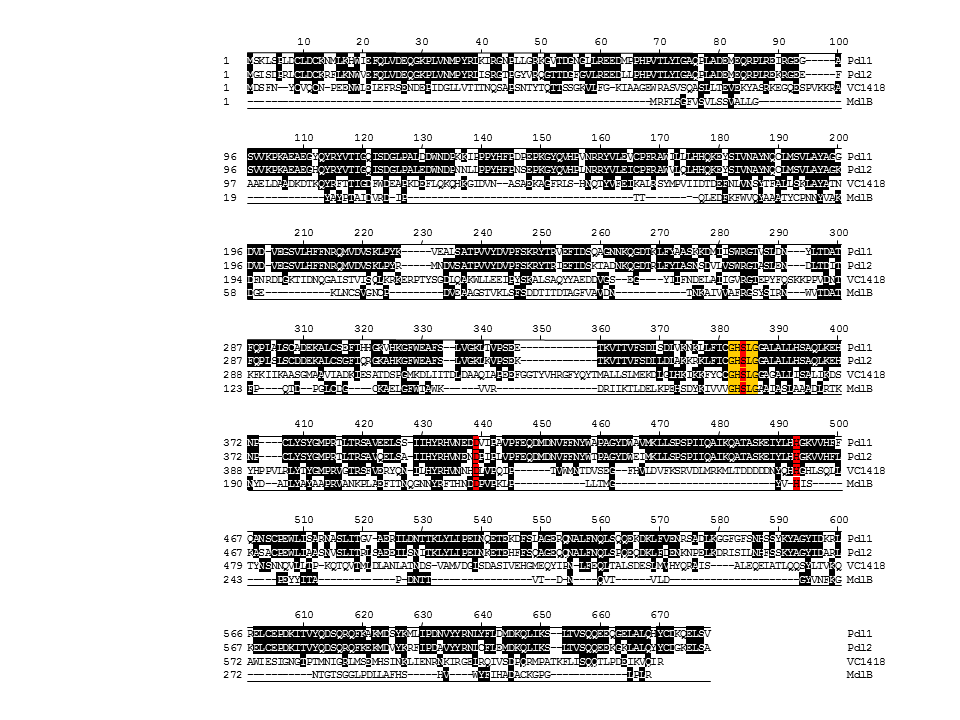

Supplement: Figure S6 — Pdl has potential protease and lipase domains. Alignment of the predicted amino acid sequences of Pl W14 Pdl1 and Pdl2 (Genbank AY144119), with predicted products of the A. oryzae mdlB gene (Genbank D85895) and a V. cholerae hypothetical open reading frame, VC1418 (Genbank AE004220). The presence of the presumptive serine protease-like catalytic triad (S, D and H) is highlighted (red) alongside the conserved pentapeptide GHSXG (yellow) common to lipases and lipoprotein lipases. (TIF) [file ppat.1002692.s006.tif]

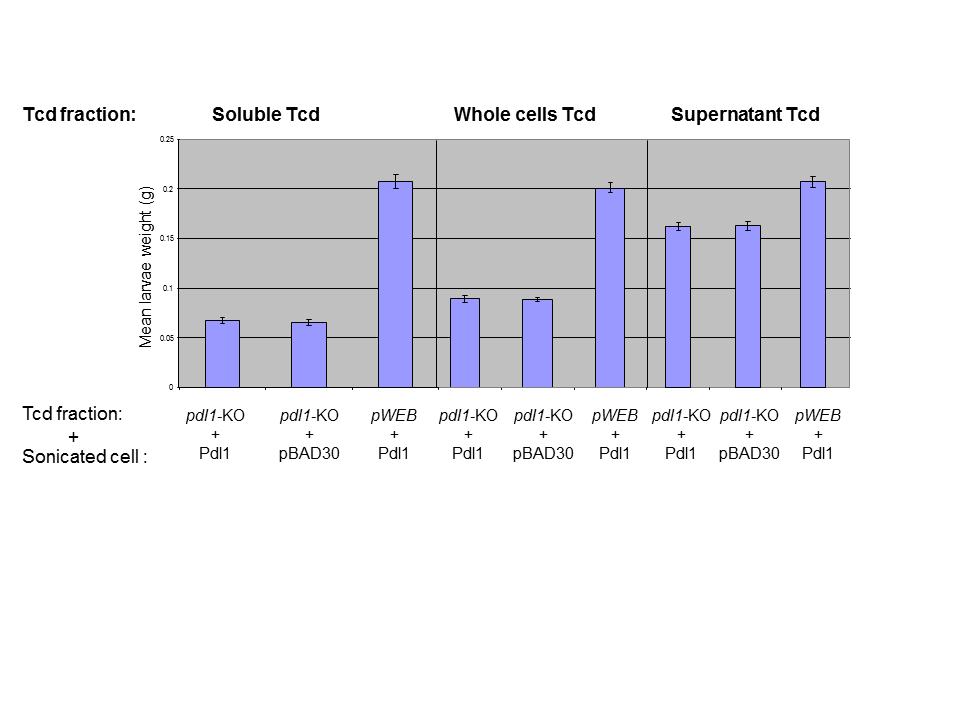

Supplement: Figure S7 — Pdl1 has no direct effect on the activity of Tcd. Mean weight gain of cohorts of M. sexta neonates (n = 10) fed different Tcd containing cell fractions (soluble, washed whole cells or supernatants) which had been pre-incubated for 1 h at 28°C with sonicated cell extracts from either an induced E. coli pBAD30-pdl1 expression construct (Pdl1) or an E. coli pBAD30 negative control (pBAD30). The Tcd fractions were isolated from the E. coli pdl1 knock out cosmid strain (pdl1-KO). We also used E. coli pWEB fractions as a further negative control (pWEB). Standard error bars are shown. The more potent the toxic effect, the smaller the mean larval weight. Note the presence of added Pdl1 does not increase toxic activity of Tcd from any fraction. Data from a 7 day assay. (TIF) [file ppat.1002692.s007.tif]

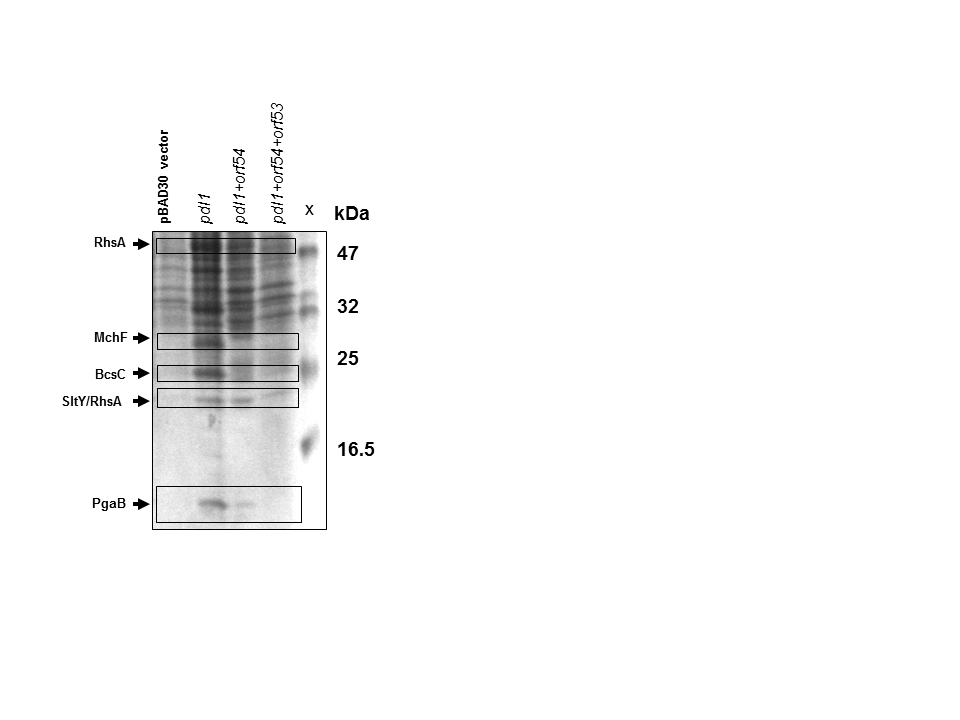

Supplement: Figure S8 — The effects of Pdl1 and Orf53 over-expression on native protein release in E. coli . The effect of pdl1, pdl1+orf54 and pdl+orf54+orf53 pBAD30 expression constructs on supernatant proteins released by the recombinant E. coli. All genes have their native Shine-Dalgarno sequences. Size markers are also shown (x). Note Pdl1 induces the release of several specific protein species (boxed). The inclusion of orf54 and orf53 (which are homologues of one another) reduce this effect in an additive manner. Putative MALDI-ToF identification of several of these E. coli protein species are indicated. (TIF) [file ppat.1002692.s008.tif]

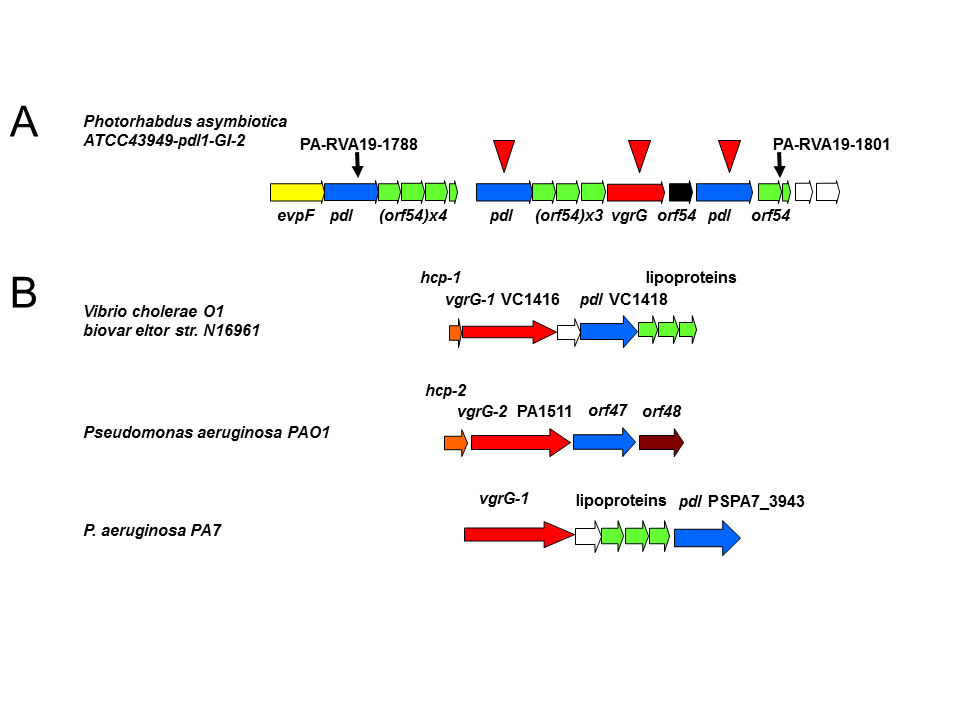

Supplement: Figure S9 — Pdl homologues are associated with other toxin secretion genes in diverse bacteria. (A) A pdl-orf54 island of P. asymbiotica ATCC43949 identified using RVA screening exhibiting insect toxicity on injection. Colour coding identifies homologous genes. Genbank locus tag numbers are given. The pdl and vgrG homologues were shown to be responsible for the toxicity of the Pa pdl-GI_2 virulence island were mapped by transposon mutagenesis (red inverted triangles) (B) pdl-orf54 homologues are often tightly linked to other toxin secretion systems in diverse pathogens such as type VI secretion systems in Vibrio and Pseudomonas. (TIF) [file ppat.1002692.s009.tif]
